# Work-family enrichment and successful aging at work: The China context

**DOI:** 10.3389/fpsyg.2022.1090864

**Published:** 2023-01-26

**Authors:** Chenhui Zhao, Huajun Ma, Zimeng Chen, Xiaohui Liu

**Affiliations:** ^1^School of Business Administration, Zhongnan University of Economics and Law, Wuhan, Hubei, China; ^2^Business School of Wuchang University of Technology, Wuhan, China; ^3^School of Management, Lanzhou University, Lanzhou, China; ^4^Business School, University of International Business and Economics, Beijing, China

**Keywords:** work-family enrichment, occupational future time perspective, successful aging at work, age-inclusive human resource practices, Chinese context

## Abstract

Existing research mainly analyzes the antecedents of successful aging at work from the perspective of the work field, ignoring that in the Chinese context of “familism,” the two fields of family and work permeate each other and may have an impact on successful aging at work. Thus, through a multi-time data collection approach, we obtained a sample of 338 older Chinese employees to examine the impact of work-family enrichment on successful aging at work, the mediating role of occupational future time perspective, and the moderating role of age-inclusive human resource practice. Results indicate that work-to-family enrichment was positively associated with successful aging at work through the mediation of occupational future time perspective. Family-to-work enrichment was positively associated with successful aging at work through the mediation of occupational future time perspective. In addition, age-inclusive human resource practice amplified the positive effects of work-to-family enrichment and family-to-work enrichment on occupational future time perspective. This is an exploration of successful aging at work in the Chinese context, broadening the theoretical research on successful aging at work and providing new ideas for managers on motivating older employees to achieve successful aging at work.

## 1. Introduction

With the intensification of global population issues such as aging and low birth rate, China’s labor force’s age structure is getting older (Feng et al., 2019). The increased number of older workers means the pressure for organizations to develop and utilize this group is becoming stronger. Besides, it is increasingly important to speed up the development of an inclusive and suitable human resource management system for older employees (Barakovic Husic et al., 2020). Successful aging at work (SAW) has attracted wide attention from scholars in China and abroad since it is an important way to develop and utilize the elderly labor force in organizations actively. The realization of SAW is the road map to making a difference for older employees and is an important prerequisite for older employees to maintain increased quality standards (Taneva and Yankov, 2020). Therefore, scholars have explored the influencing factors of SAW from the individual and work areas (Hanscom and Cleveland, 2018). Individual aspects such as proactive personality (Zacher et al., 2018), personal resources (Kooij et al., 2014), work areas such as job characteristics, work willingness perception (Taneva et al., 2014). But the individual’s life area includes not only the work area, but also the family area, especially in China where “family culture” is prevalent. The family area is also important (Lam et al., 2012). Therefore, it is not sufficient to explore the reasons for employee behavior from any of the two fields alone (Zhang et al., 2018), and it is necessary to consider the influence of mutual penetration between the two fields. There are few discussions on the Chinese context. There are potential differences between Western and Eastern societies in how individuals perceive the work-family interface (Powell et al., 2009). In the Chinese context, older employees are more willing to combine their work and family roles. Family is regarded as the basis of their whole life, and work is regarded as a tool to support the family (Jin et al., 2013; Cheung and Wu, 2013b), if the relationship between work and family is well handled, it may help older employees successfully aging at work. The relationship between work and family mainly includes negative Work-Family Conflict and positive work-family enrichment, which will encourage employees to develop negative and positive behaviors, respectively (Vieira et al., 2018; Zhang et al., 2018). Therefore, combined with the resource conservation theory, which states that resources can promote individual positive behavior, this study proves that it is necessary to analyze the reasons for the successful aging of older employees from the perspective of work-family enrichment (Halbesleben et al., 2009).

Presently, research attention has seldom paid to explore the mechanism of work-family enrichment on SAW, despite many studies acknowledging the positive impacts of work-family enrichment on employee behavior. Work→family enrichment (W-FE) and family→work enrichment (F-WE) both have a positive impact on employees’ work engagement, citizenship behavior, performance, and other behaviors (Zhang et al., 2018). However, the mechanism by which the former affects the latter has not been taken seriously. According to the socioemotional selectivity theory, with an increase in age, the choice of future goals for older employees changes significantly (Carstensen et al., 1999). They believe that the profession’s future is becoming increasingly unclear, and emotional needs are increasing. As a harbor for individual emotional sustenance, the family may play an important role in influencing the occupational future time perspective (OFTP) (Carstensen, 2006). The improvement of OFTP contributes to SAW (Cheung et al., 2017). Therefore, this study further proves that it is necessary to analyze the mediating mechanism of the impact of work-family enrichment on SAW from the OFTP.

Additionally, age-inclusive human resource practices (AIHRP) have important implications for companies to address age discrimination and age stereotypes (Boehm et al., 2013). The realization of SAW cannot ignore the influence of situational factors such as the organizational environment. This study expects that work-family enrichment can promote older employees to achieve SAW through OFTP. In this process, age-inclusive human resource practices contribute to the effectiveness of work-family enrichment and OFTP. Presently, few studies have explored the effect of age-inclusive human resource practices on work-family enrichment as this study is aimed at doing.

**FIGURE 1:**
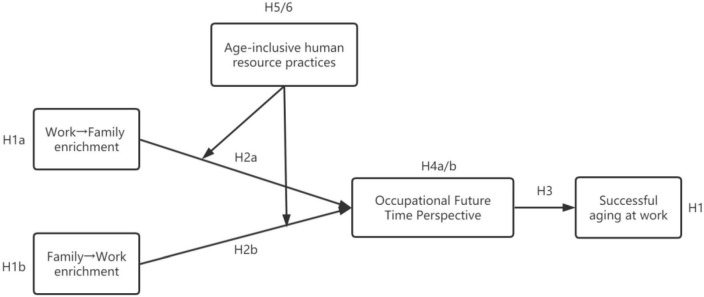
Conceptual model.

According to Figure 1, this study hopes to understand how work-family enrichment affects SAW in the Chinese context and the corresponding intermediate mechanisms and boundary conditions. The study is expected to expand the antecedents of successful aging in older employees’ work through in-depth research and answers to scheduled questions and provide new ideas for managers on motivating older employees to achieve SAW.

## 2. Theory development

Based on the logic of “resource-select-behavior,” the conservation of resources theory (COR) and socioemotional selectivity theory (SST) support the theory of development. COR suggests that individuals tend to put existing resources into use to recover from resource consumption or to acquire new resources (Halbesleben et al., 2009). Following this logic, this study suggests that employees will use resources obtained from the work and family domains to invest in the resource-consuming behavior of SWA because once SAW is achieved, employees may have access to more resources (Burris, 2012). At the same time, due to the bidirectional nature of work-family enrichment, the different resources obtained by older employees in these two directions may promote the SAW of older employees. Besides, the SST holds that individuals choose their goals according to their limited or unlimited perception of the future (Carstensen et al., 1999). For example, goals that help in the future, such as acquiring new knowledge or improving skills, are prioritized when time is considered infinite. Contrarily, if time is limited, short-term interests such as emotional appeals will be prioritized. The resources acquired by older employees from work-family enrichment may be used to meet the needs of working hours and promote OFTP. That is, OFTP may be the intrinsic reason why work-family enrichment affects the SAW (Choi et al., 2018). Finally, this study introduced AIHRP as an important contextual factor, providing boundary factors for work-family enrichment affecting OFTP (Boehm et al., 2013).

## 3. Hypotheses development

### 3.1. Work-family enrichment and SAW

The concept of successful aging originated from gerontology, meaning that the elderly can show low prevalence, have the high cognitive ability and physical function and adapt well to the social environment (Rowe and Kahn, 1997). After that, this concept is extended to the organization. Successful aging at work broadly refers to the attainment, growth, or maintenance of favorable work outcomes with increasing age (Kooij, 2015; Zacher, 2015; Zacher et al., 2018). Scholars have expounded on the connotation of SAW from different perspectives (Zacher et al., 2018; Kooij et al., 2020). From a career perspective, SAW is reflected in being able to adapt to changes in their bodies and work, positive interpersonal relationships, better career development in the future, personal safety guaranteed, and continuous realization of their own life goals (Robson et al., 2006). From a maintenance perspective, SAW is defined as the ability of older employees to maintain a healthy level of motivation and ability to work (Kooij et al., 2015). From a comparative perspective, SAW refers to older employees who can show higher levels of work than their peers (Zacher, 2015). Although scholars have different focuses, SAW shows the positive cognition of older employees on themselves and their working environment from any perspective (Taneva and Yankov, 2020). Therefore, the author synthesizes the viewpoints of previous studies and the measurability of concepts and defines SAW as a state in which older employees can show positive cognition of their age and high adaptability to changes in the external environment.

Work-family enrichment refers to the positive aspect of the infiltration between work and family. It is a two-way concept, including two dimensions of work-family enrichment (W-FE) and family-work enrichment (F-WE) (Heskiau and McCarthy, 2021). Among them, work-to-family enrichment refers to the accumulation of resources in the field of work that can improve the family support quality of life; for example, achievements at work may produce a positive mood, promoting family harmony and life satisfaction. Family-to-work enrichment refers to the resource support accumulated in the family field that can improve the quality of work (Zhang et al., 2018). In China, family roles and work roles frequently intertwine. It is not enough to explore the antecedents of the successful aging of older employees only from the work field. Hence, it is necessary to consider the dual effects of family and work roles comprehensively.

The study found that resources have a significant predictive effect on successful aging at work (Zacher et al., 2018), and work-family conflict has a negative impact on SAW (Cheung and Wu, 2012), because when work-family conflict occurs, older employees will devote more resources to family roles rather than work areas, which will make older employees unable to focus on work tasks and hinder further career development. This is because for older employees, family is more important, especially in the Chinese context, where career or work roles are not the core role of older employees, and family roles are the most important (Cheung and Wu, 2012, 2013b). Work-family enrichment may contribute to the realization of SAW for older employees, because work-family enrichment means the expansion of resources and contributes to positive results in the work field. Studies have found that work-family enrichment or family-work enrichment is positively correlated with work engagement (Gali Cinamon and Rich, 2009; Timms et al., 2015). However, there is no research on the relationship between work-family enrichment and SAW, so we explore the relationship between work-family enrichment and SAW.

According to the COR, work-family enrichment can be understood as a process in which resources from both work and family fields promote the overall increase of individual resources and form a “gain spiral” (Hobfoll, 2002). Therefore, resources in both fields promote the increase of resources in other fields; that is, the resources of older employees at work increase, and the realization of SAW becomes possible. The higher the W-FE, the easier it is for older employees to achieve successful aging at work. On the one hand, the higher the W-FE is, the more resources older employees can obtain from work to meet their family needs (Carlson et al., 2010), which are conducive to older employees meeting their family responsibilities (Zhang et al., 2018). When older employees perceive that the resources they obtain from work help them meet the needs of their families, they will appreciate the support of the organization (McNall et al., 2010), which helps to enhance the positive emotions and work adaptability of employees (Srivastava et al., 2006). In the Chinese context, families are more sensitive to the influence of older employees (Cheung and Wu, 2012), which helps to enhance the positive emotions and work adaptability of older employees (Cheung and Wu, 2012), and encourage older employees to achieve successful aging at work. Therefore, the study predicts that W-FE will positively impact the SAW of older employees. On the other hand, the higher the F-WE, the easier it is for older employees to achieve successful aging at work. Because the higher the F-WE, the more resources the family domain penetrates into the work field (Carlson et al., 2010). These excess resources help older employees to effectively cope with stress (Carlson et al., 2011), improve the adaptability of older employees, and help older employees achieve successful aging at work. These resources can encourage older employees to meet their in-role behavior and work needs and realize SAW. As such, the following hypotheses were developed.

**H1**: WFE is positively related to the SAW of older employees.

**H1a**: W-FE is positively related to the SAW of older employees.

**H1b**: F-WE is positively related to the SAW of older employees.

### 3.2. Mediation effect of OFTP

OFTP refers to employees’ perception of their occupation future in the working environment (Henry et al., 2018). Zacher and Frese combined the connotation of FTP with the working environment (Zacher and Frese, 2009), and developed the OFTP scale based on the FTP scale (Carstensen and Lang, 1996). Specifically, OFTP refers to employees’ perceptions of their own career remaining time and opportunities (Zacher and Frese, 2009), including two dimensions of perceived remaining time and focus on opportunities, both of which are negatively correlated with age (Zacher and Frese, 2009; Froehlich et al., 2016). Similar to FTP effects (Kooij et al., 2018), OFTP also plays an important role in work (Henry et al., 2018), and a large number of studies have shown that OFTP has a positive relationship with important work-related outcomes (Rudolph et al., 2018). Such as work engagement, work attitude (Henry et al., 2018). Further, Topa and Zacher developed the Spanish OFTP scale (perceived remaining time, focus on opportunities, focus on limitations), which found that OFTP can assess the cognitive and emotional expression of older adults regarding the remaining time associated with work (Topa and Zacher, 2018). Therefore, it is important to determine the variables that predict OFTP to increase the level of OFTP, thereby revealing how to improve the beneficial results. Henry and Desmette show that work-family conflict has a negative impact on OFTP, and through the role of OFTP, it reduces the effective strategies of individuals to solve related problems (Henry et al., 2018). At present, there are few studies on the relationship between work-family enrichment and OFTP. Only Henry and Desmette have explored the relationship between work-family enrichment and OFTP, and found that W-FE has a positive relationship with perceived residual time dimension, and this effect is more obvious in older groups. In addition, Alcover et al.’s research further emphasized the important mediating role of OFTP between work-family relationships and work outcomes, and found that two-way interference between work and family affects employees’ willingness to retire through OFTP (Alcover et al., 2022). As far as we know, there is no research to explore the mediating role of OFTP in the relationship between work-family gain and SAW. Studies have found that two-way work-family enrichment increases individual resources (Mishra et al., 2014, 2019), therefore, we believe that W-FE, F-WE contribute to OFTP improvement.

According to the meaning of W-FE and F-WE, the higher the W-FE, the more favorable it is for employees to obtain resources to meet family needs from the work field (Schmitt et al., 2012). The higher the W-FE, the more beneficial it is for older employees to obtain family resources from work to meet family needs (ten Brummelhuis and Bakker, 2012). When the need for family support is met, older employees will form a resource-gain spiral (Hobfoll, 2002), and family stress is reduced, helping them gain more resources (ten Brummelhuis and Bakker, 2012) that may help older employees see more time and opportunities. Similarly, the higher the F-WE, the more favorable it is for employees to obtain resources from the family to meet their work needs. Higher F-WE means that older employees can obtain more resources to support their work from the family field and see more remaining time (Rudolph et al., 2018). In addition, older employees may also obtain valuable skills or flexible working methods from the family field (Balmforth and Gardner, 2006), which helps them focus on more job opportunities. Older employees will meet the resources needed in both fields through the “income spiral” from work and family resources (Ho and Yeung, 2016). This ensures that the needs of both work and family fields are met accordingly. Reducing the time and energy consumption of older employees is conducive to their optimistic grasp of the remaining time and opportunities in their careers, and their OFTP is more confident, and OFTP becomes more open. As such, the following hypotheses were developed.

**H2**: WFE is positively related to the OFTP of older employees.

**H2a**: W-FE is positively related to the OFTP of older employees.

**H2b**: F-WE is positively related to the OFTP of older employees.

We propose that OFTP is helpful to the realization of SAW. There is little exploration of the relationship between OFTP and SAW. Only one manuscript analyzes the relationship between OFTP and SAW (Cheung et al., 2019), and finds that OFTP is positively correlated with SAW. When older employees have high OFTP, they will set more goals for their careers and are eager to acquire new knowledge and skills (Ho and Yeung, 2016). According to the SST, employees with high OFTP tend to use positive problem-solving strategies when dealing with work problems. In contrast, employees with low OFTP tend to use passive coping strategies when dealing with the same problems (Cheung et al., 2017). We believe that the OFTP of older employees contributes to the realization of SAW because when older employees think they have an open OFTP, they will set long-term goals for their career future, focus on the realization of goals, and have a strong motivation to learn work-related knowledge and skills, improve their ability to solve work problems, and show a positive cognitive level and high adaptability (Weikamp and Göritz, 2016). When older employees have high OFTP, they will set more goals for their careers and are eager to acquire new knowledge and skills (Ho and Yeung, 2016), which helps them have higher adaptability in their positions. In addition, high OFTP older employees. They also have a positive view of changes in age because they will think they have a long time to go before leaving their position or organization. As such, the following hypothesis was developed.

**H3**: OFTP is positively related to the SAW of older employees.

Furthermore, we believe that OFTP is a potential influence mechanism of family-work enrichment on the SAW of older employees (Zacher, 2013). This is because whether it is W-FE or F-WE, it can effectively improve the personal resources of older employees. Only when the resources they have can meet the “in-role things” of work and family will older employees invest excess resources in expanding OFTP’s “out-role things” (Halbesleben et al., 2014). Therefore, the satisfaction of work and family needs is conducive to the improvement of OFTP. Additionally, older employees with high OFTP will strive to achieve long-term goals, eager to acquire new work knowledge and skills, improve work adaptability, establish a broad social network, and continue to focus on work (Zacher and Rudolph, 2019). They will also show high cognitive ability and adaptability to achieve SAW. According to the above assumptions, H2, H2a, and H2b show that work-family enrichment positively impacts OFTP. H3 and this study show that OFTP has a positive impact on SAW. Taken together, OFTP plays a mediating role between work-family enrichment and SAW. Therefore, we propose the following hypotheses.

**H4**: OFTP mediates the relationship between WFE and SAW of older employees.

**H4a**: OFTP mediates the relationship between W-FE and SAW of older employees.

**H4b**: OFTP mediates the relationship between F-WE and SAW of older employees.

### 3.3. Moderating role of AIHRP

Older employees accumulate rich resources and energy for themselves through work-family enrichment. Still, the effect of using the resources differs, depending on how much external support they can obtain. Therefore, we try to use AIHRP as an important amplifier to promote OFTP by building a fair and just human resource management atmosphere, releasing the beneficial resources of work-family enrichment.

AIHRP refers to specific practical activities in which the organization integrates age-inclusive culture and concept into human resource management elements such as policies, means, and systems that affect employee behavior, attitudes, and performance (Boehm et al., 2013). It is reflected in the inclusive, fair, non-discriminatory, and non-prejudiced treatment of employees of all age groups (Oliveira and Cabral-Cardoso, 2018). Employees of all ages will receive the same level of support from the organization, with equal access to training, skills improvement, and promotion opportunities. This way, the organization can ensure that employees of different ages have the necessary knowledge and skills to work, complete organizational tasks, and ultimately promote organizational performance (Liu, 2018). Compared with traditional human resource practices, AIHRP pays more attention to the impact of age on employees’ work behavior, works to eliminate the stereotypes caused by age, helps stimulate the work vitality of employees of different ages, and enhances the work motivation of older employees, with inclusive, fair, non-discriminatory values and practical spirit (Boehm et al., 2013). AIHRP provides older employees with a sense of organizational support, reduces the negative impact of age on employees, and provides a broad platform and support for older employees to give full play to their premise. Existing research has found that human resource practices contribute to the enrichment of resources, thereby increasing work-family enrichment (Greenhaus and Powell, 2006), and that human resource practices have a significant positive effect on work-family enrichment (Chen et al., 2018). However, no research has explored whether age-inclusive human resource practices also play a catalytic role in this path of work-family enrichment affecting OFTP. More specifically, we believe that age-inclusive human resource practices amplify the positive relationship between work-family enrichment and OFTP. As mentioned above, work-family enrichment can improve OFTP through the benefit spiral of resources. However, the impression of age bias and age discrimination in the organization may interfere with the use of resources by older employees (Maurer et al., 2003; Ng and Feldman, 2012; Akkermans and Kubasch, 2017). AIHRP can reduce age-based stereotype threats in the workplace (Oliveira and Cabral-Cardoso, 2018) and can support older employees’ motivation for work (Taneva and Arnold, 2018). Because AIHRP creates an age-inclusive environment, it provides a sense of organizational support for older employees, reduces discriminatory problems, and enables older workers to fully realize their potential (Templer et al., 2010). The high level of age-inclusive human resource practices supply more positive emotions and supportive signals to older employees. When older employees feel that the organization is taking care of them, for example through AHIRP, it will enable older employees to have a positive view of future opportunities and time (Fasbender et al., 2019), which in turn promotes the impact of work-family enrichment on OFTP.

If the family-to-work gain can be achieved, it means that they have generated favorable resources in the family domain. Family experience can improve the quality of another work experience. AIHRP can amplify the effect of this experience so that older employees can access family domain resources and apply them to work tasks smoothly without being affected by age discrimination or stereotypes (Schmitt et al., 2012). On the other hand, when work-to-family enrichment can be achieved, it means that the resources obtained by older employees from work can promote the improvement of family life quality. According to the COR, improving family resources can improve the acquisition of resources in the work field. AIHRP is conducive to the further transformation of family resources into work resources. Older employees have sufficient external support for resource allocation and will not be affected by age. Organizational support ensures that older employees can see more opportunities and time, and OFTP increases.

Further, we believe that the mediating role of OFTP depends on the degree of implementation of AIHRP. When the organization implements strong AIHRP, F-WE and W-FE have a more positive impact on the OFTP of older employees, promoting the realization of SAW of older employees. On the contrary, when the organization implements weak AIHRP, this impact will be weakened.

**H5**: AIHRP moderates the positive effects of WFE on OFTP, such that these effects are stronger for organizations with high (vs. low) AIHRP.

**H6**: AIHRP moderates the indirect effects of WFE on SAW via OFTP, such that these indirect effects are stronger for organizations with high (vs. low) AIHRP.

## 4. Materials and methods

### 4.1. Participants and procedures

All participants were Chinese full-time employees aged 45 or above. The age of 45 is the cut-off age defined by the World Health Organization, which means the end of youth. The World Health Organization (WHO) defines employees aged 45 and above as “older workers” (World Health Organization, 1993). In addition, the study found that employees over the age of 45 began to experience a significant decline in their physical conditions and that employee behavior and job performance would begin to change from the age of 45 (Kulik et al., 2016). Therefore, this study sets the age standard for older employees at 45 years or above.

Employees of a large service company in China were asked to complete a self-reported questionnaires. Due to busy work, it is difficult to contact employees without personal recommendation. The data collection method is mainly offline, and online collection is assisted. The management has formally approved the receipt collection of each organization. The author communicated with them during their work and told them the purpose of the study. The questionnaire is issued and collected only for employees who voluntarily participate in the research. In the questionnaire survey process, to ensure the interviewees’ privacy, the questionnaire is filled in anonymously. The purpose and precautions of the questionnaire survey are informed to ensure the validity and objectivity of the data. The questionnaire is distributed in multiple periods to reduce the common method deviation and matched by the last four digits of the employee’s mobile phone tail number. The author visited them in the first period to collect information about W-FE, F-WE, and control variables. After 30 days, the author visited the same employees and collected information about OFTP and SAW. After waiting for 30 days, the author collected information about AIHRP.

The respondents were required to fill in the last four digits of their mobile numbers, which would late be compared to determine whether they matched. In the first stage of the questionnaire, 370 questionnaires were distributed, and 354 questionnaires were recovered. After 30 days, 354 respondents were contacted to collect the data for the second study, and 350 questionnaires were recovered. After 30 days, questionnaires were distributed to 350 respondents, and 341 questionnaires were collected. After mobile phone number matching, 338 questionnaires were collected for data analysis. The questionnaires showed that 45.6% were women and 54.4% were men. The average age of the sample was 51.8 years old, of which 176 were 45–49 years old, accounting for 52.07%; 115 people aged 50–54, accounting for 34.02%; 32 people aged 55–59, accounting for 9.47%; 15 people aged 60 and above accounted for 4.43%. In terms of years of work, 78.40% of respondents have 21–30 years of work experience and 6% have more than 30 years of work experience of the 338 respondents, 45.6% were female and 54.4% were male. The majority of the respondents were between 50 and 54 years old (54.14%), followed by 45–49 years of age (20.41%), 55–60 years of age (18.93%) and over 60 years old (6.51%). 41.12% of employees had an HSSC, 27.51% had bachelors’ degree, 26.62% had Master degree and 4.73% were MS/Ph.D. In terms of years of work, 78.40% of respondents have 21–30 years of work experience and 6% have more than 30 years of work experience (Kim et al., 2017).

### 4.2. Measures

#### 4.2.1. Work-family enrichment

Work-family enrichment is a two-way variable that includes W-FE and F-WE. A scale developed by Kacmar measures it; each dimension contains three items (Kacmar et al., 2014). For example, W-FE “The skills I learned at work make me better interact with family members,” F-WE “The harmony of family life makes me more confident in my work.” The questionnaire was measured by the Likert Five-point measures. The Cronbach’s α coefficient of W-FE was 0.897, and the Cronbach’s α coefficient of F-WE was 0.905.

#### 4.2.2. OFTP

To measure OFTP, the author adopts the scale of Zacher, which contains six items (Zacher and Frese, 2009). The items include “I think my career future is infinite” and so on. Cronbach’s α was 0.913.

#### 4.2.3. SAW

To measure SAW, the author uses Taneva’s scale, a total of 20 items (Taneva and Yankov, 2020). For instance, “I can quickly adapt to changes at work”. For SAW value of Cronbach’s coefficient α was 0.921.

#### 4.2.4. AIHRP

To measure AIHRP, the author uses Boehm’s scale, a total of 5 items, including “Does your company offer equal opportunities to be promoted, transferred, and to make further career steps irrespective of one’s age?” and so on (Boehm et al., 2013). For AIHRP, Cronbach’s alpha coefficient comes out to be 0.917.

#### 4.2.5. Control variables

The author takes gender, age, education, and working years as control variables. The classification criteria were (1 = male; 2 = female); age (1 = 45–49 years old; 2 = 50–54 years old; 3 = 55–59 years old; 4 = 60 years old and above); education (1 = HSSC; 2 = Bachelor’s; 3 = Master degree; 4 = MS/Phil and PhD); working years (1 = 10–20 years, 2 = 21–30 years, 3 = 31–40 years, 4 = 41 years and above). In previous studies, these demographic variables are related to the behavior of older employees, so these variables are used as control variables to more accurately study the influence mechanism of family-work enrichment on the SAW of older employees (Schwall, 2012).

## 5. Results

### 5.1. Common method variance

The author first carried out Harman single-factor test to avoid common method bias before data analysis. Tests showed that a common method factor explained 31.6% of the total variance in the results (31.6% < 40%). In addition, data items used were placed on a common factor for model fitting, and the fitting results were χ^2^/df = 6.9380, CFI = 0.473, TLI = 0.471, RMSEA = 0.146, SRMR = 0.121. The fitting situation was poor, indicating that the common method bias was not serious. Following Richardson’s recommendations (Richardson et al., 2009), the authors used the ULMC method (Unmeasured Latent Method Construct) to test common method biases. The analysis results show that before the common method factor is put into the five-factor model, the fitting results are χ^2^/df = 1.4956, CFI = 0.931, TLI = 0.927, RMSEA = 0.063, SRMR = 0.057. After adding the common method factor to the four-factor model, the fitting results are χ^2^/df = 1.5017, CFI = 0.928, TLI = 0.921, RMSEA = 0.069, SRMR = 0.062. The fitting results have not been greatly improved, indicating that the common method bias of this study is not serious.

### 5.2. Confirmatory factor analysis

We use the maximum likelihood method to evaluate the discriminant validity of CFA model parameters. This includes checking the values of model fit indices such as CFI, TLI, and RMSEA. The test results are shown in Table 1. The results showed that the five-factor model was good, χ^2^ = 1087.312, df = 727, χ^2^/df = 1.4956, CFI = 0.931, TLI = 0.927, RMSEA = 0.063, SRMR = 0.057.

**TABLE 1:** Results of the confirmatory factor analysis (*N* = 338).

Model	χ^2^	df	χ^2^/df	CFI	TLI	RMSEA	SRMR
Five-factor model: W-FE, F-WE, OFTP, AIHRP, SAW	1,087.312	727	1.4956	0.931	0.927	0.063	0.057
Four-factor model: W-FE, F-WE, OFTP, AIHRP+SAW	2,153.638	732	2.9421	0.867	0.851	0.083	0.079
Three-factor model: W-FE, F-WE, OFTP+AIHRP+SAW	3,148.275	733	4.2951	0.779	0.753	0.102	0.097
Two-factor model: W-FE, F-WE+OFTP+AIHRP+SAW	4,172.752	736	5.6695	0.573	0.564	0.137	0.105
One factor model: W-FE+F-WE+OFTP+AIHRP+SAW	5,113.326	737	6.9380	0.473	0.471	0.146	0.121

**χ**^2^, normal-theory weighted least-squares Chi square; TLI, tucker–lewis fit index; CFI, the comparative fit index; RMSEA, the root-mean square error of approximation; SRMR, the standardized root-mean-square residual; W-FE, work→family enrichment; F-WE, family→work enrichment; OFTP, occupational future time perspective; AIHRP, age-inclusive HR practices; SAW, successful aging at work.

Table 2 shows that W-FE is positively correlated with OFTP (*r* = 0.432, *p* < 0.01) and SAW (*r* = 0.207, *p* < 0.01). Family-to-work gain is positively correlated with OFTP (*r* = 0.451, *p* < 0.01) and SAW (*r* = 0.312, *p* < 0.01). OFTP is positively correlated with SAW (*r* = 0.391, *p* < 0.01). The analysis results preliminarily support the hypothesis.

**TABLE 2:** Mean, SD, correlations, and reliability.

Variables	Mean	SD	1	2	3	4	5	6	7	8	9
1. Gender	1.423	0.474									
2. Age	2.137	0.688	-0.031								
3. Education	1.761	0.653	0.034	0.002							
4. Experience	2.174	0.718	-0.071	0.026	0.043						
5. W-FE	3.153	0.845	0.013	0.012	-0.014	0.051	**(0.897)**				
6. F-WE	3.271	0.827	0.045	0.024	0.005	0.017	0.473**	**(0.905)**			
7. OFTP	2.782	0.709	0.036	-0.042	0.017	0.009	0.432**	0.451**	**(0.913)**		
8. SAW	2.354	0.765	0.018	0.027	0.046	0.022	0.207**	0.312**	0.391**	**(0.921)**	
9. AIHRP	1.816	0.772	-0.031	-0.005	0.032	0.031	0.101**	0.115**	0.138**	0.217**	**(0.917)**

**Correlation is significant at the 0.01 level (2-tailed). The bold values indicated the cronbach’s alpha. W-FE, work→family enrichment; F-WE, family→work enrichment; OFTP, occupational future time perspective; AIHRP, age-inclusive HR practices; SAW, successful aging at work.

### 5.3. Results for direct and indirect effect

In Table 3, W-FE is positively correlated with OFTP (β = 0.237, *p* < 0.01), W-FE is positively correlated with SAW (β = 0.197, *p* < 0.01), supporting H1, H1a and H2a. F-WE was positively correlated with OFTP (β = 0.254, *p* < 0.01), F-WE was positively correlated with SAW (β = 0.203, *p* < 0.01), supporting the hypothesis of H2, H2a and H2b. OFTP was positively correlated with SAW (β = 0.316, *p* < 0.01), H3 was supported. In addition, the author tested the mediating effect by Monte Carlo method. By constructing the structural equation model, 5000 Bootstrapping samples were extracted to verify the mediating effect of OFTP between W-FE, F-WE and SAW. The results are shown in Table 4. The mediating effect of OFTP between W-FE and SAW is 0.374, 95% CI [0.094, 0.186], and the confidence interval does not contain zero, indicating that the mediating effect exists and supports hypothesis H4a. The mediating effect of OFTP on the relationship between F-WE and SAW was 0.431, 95% CI [0.105, 0.218], and the confidence interval did not contain zero, indicating that the mediating effect existed, supporting the hypothesis H4b, and the hypothesis H4 was also supported.

**TABLE 3:** Direct effect.

Direct effects	β	SE	*t*	*p*
W-FE→OFTP	0.237	0.056	4.232	0.000
W-FE→SAW	0.197	0.048	4.104	0.000
F-WE→OFTP	0.254	0.054	4.703	0.000
F-WE→SAW	0.203	0.051	3.980	0.000
OFTP→SAW	0.316	0.062	5.097	0.000

*N* = 338. W-FE, work→family enrichment; F-WE, family→work enrichment; OFTP, occupational future time perspective; SAW, successful aging at work.

**TABLE 4:** Indirect effect.

Path’s effects	β	SE	LL95%	UL95%
W-FE→OFTP→SAW	0.374	0.025	0.094	0.186
F-WE→OFTP→SAW	0.431	0.019	0.105	0.218

W-FE, work→family enrichment; F-WE, family→work enrichment; OFTP, occupational future time perspective; SAW, successful aging at work.

### 5.4. Moderation analysis

**FIGURE 2:**
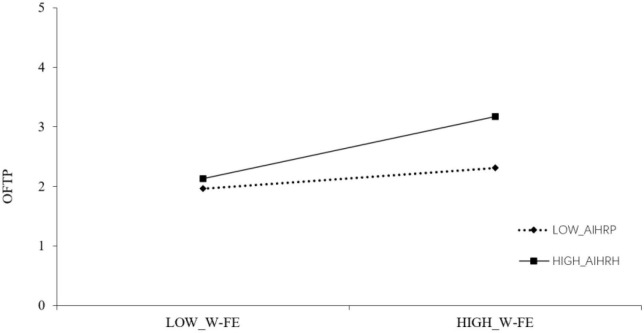
The moderating role of AIHRP between WF-E and OFTP. W-FE, work→family enrichment; OFTP, occupational future time perspective; AIHRP, age-inclusive HR practices.

**FIGURE 3:**
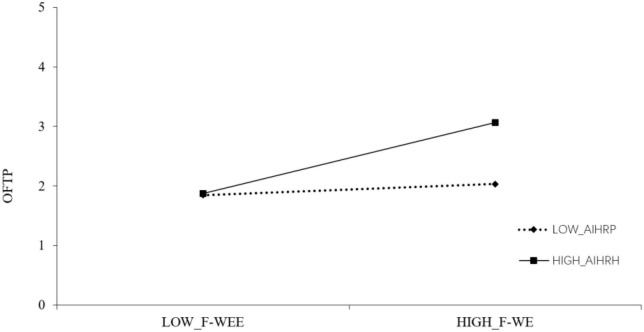
The moderating role of AIHRP between F-WE and OFTP. F-WE, family→work enrichment; OFTP, occupational future time perspective; AIHRP, age-inclusive HR practices.

The author gives the test results of the moderating effect in Tables 5, 6. It can be seen that the interaction term of W-FE and AIHRP is significantly positively correlated with OFTP (β = 0.1742, SE = 0.0577, *p* < 0.01). In order to test this interaction effect, the author draws the corresponding moderating effect diagram in Figure 2. When AIHRP is higher than a standard deviation, the relationship between W-FE and OFTP is more positive and significant. The interaction between F-WE and AIHRP in Table 6 was significantly positively correlated with OFTP (β = 0.2517, SE = 0.0607, *p* < 0.01). In order to test this interaction effect, the author drew a moderating effect diagram in Figure 3. When AIHRP is higher than one standard deviation, the relationship between F-WE and OFTP is more positive and significant, assuming that H5 is supported.

**TABLE 5:** Moderated mediation model results.

Variable	AIHRP (W)					
	W-FE (X)		*x*		OFTP (Y)	
	β	SE	*T*	*P*	95% CI	
					LL	UL
Constant	2.3942	0.4842	4.9445	0.0000	2.0804	2.3101
W-FE	0.3478	0.0762	4.5643	0.0000	0.2086	0.3690
AIHRP	0.2575	0.0738	3.4891	0.0002	0.1074	0.2513
Interaction	0.1742	0.0577	3.0190	0.0037	0.0836	0.3749

W-FE, work→family enrichment; OFTP, occupational future time perspective; AIHRP, age-inclusive HR practices.

**TABLE 6:** Moderated mediation model results.

Variable	AIHRP (W)					
	W-FE (X)		*x*		OFTP (Y)	
	β	SE	*T*	*P*	95% CI	
					LL	UL
Constant	2.2052	0.4813	4.5818	0.0000	2.0931	2.3218
W-FE	0.3443	0.0463	7.4363	0.0000	0.1778	0.2706
AIHRP	0.2648	0.0638	4.1504	0.0000	0.1353	0.4135
Interaction	0.2517	0.0607	4.1467	0.0000	0.2358	0.5376

W-FE, work→family enrichment; OFTP, occupational future time perspective; AIHRP, age-inclusive HR practices.

### 5.5. Testing the moderated mediation effects

Next, the author studied whether AIHRP regulates the indirect effect of W-FE and F-WE on SAW through OFTP. The results are shown in Table 7 According to the Monte Carlo bootstrapping results, when AIHRP is less than a standard deviation of the mean value, the indirect effect of W-FE on SAW through OFTP is 0.103, 95% CI is [0.047, 0.101]. When AIHRP is higher than a standard deviation of the mean value, the indirect effect of W-FE on SAW through OFTP is 0.156, 95% CI is [0.093, 0.225]. The confidence interval does not contain zero, and the difference is significant, 95% CI [0.021, 0.069]. The confidence interval does not contain zero; when AIHRP is less than a standard deviation of the average value, the indirect effect of F-WE on SAW through OFTP is 0.108, 95% CI is [0.053, 0.109]. When AIHRP is higher than a standard deviation of the average value, the indirect effect of F-WE on SAW through OFTP is 0.215, 95% CI is [0.182, 0.317], the confidence interval does not contain zero, the difference is significant, 95% CI [0.064, 0.035], the confidence interval does not contain zero, assuming that H6 is supported.

**TABLE 7:** Results of the moderated mediation.

AIHRP	Indirect effect	SE	LL95%	UL95%
**W-FE→OFTP→SAW**				
Low (mean – 1 SD)	0.103	0.041	0.047	0.101
High (mean + 1 SD)	0.156	0.049	0.093	0.225
High vs. low	0.054	0.012	0.021	0.069
**F-WE→OFTP→SAW**				
Low (mean – 1 SD)	0.108	0.032	0.053	0.109
High (mean + 1 SD)	0.215	0.051	0.182	0.317
High vs. low	0.097	0.025	0.064	0.135

W-FE, work→family enrichment; F-WE, family→work enrichment; OFTP, occupational future time perspective; AIHRP, age-inclusive HR practices; SAW, successful aging at work.

## 6. Discussion

The authors studied the effects of W-FE and F-WE on SAW, the mediating role of OFTP, and the moderating role of AIHRP. According to the research results, W-FE, F-WE, and OFTP are positively correlated. W-FE often shows that older employees can obtain resources from the work field to meet the needs of their families, which enhances the positive emotions of older employees at work and is conducive to the openness of their OFTP. F-WE often shows that the resources older employees receive from their families are conducive to the development of their work. In the Chinese context, support from the family is the driving force for the efforts of older employees, and OFTP also increases. Consistent with previous studies, OFTP contributes to the realization of SAW for older employees, which is consistent with previous studies and supports SST (Cheung et al., 2019). This theory holds that when individuals have open OFTP, they will be motivated to acquire knowledge and skills and improve their positive cognition of age and adaptability to environmental changes. In addition, this study also explored the positive relationship between W-FE, F-WE, and OFTP regulated by AIHRP and the indirect relationship between W-FE, F-WE, and SAW through OFTP.

### 6.1. Theoretical implications

There are five main theoretical implications of this study. Firstly, because different cultures have different influence on successful aging, it is very important to analyze successful aging in a specific cultural background. Compared with studies on successful aging at work in Western cultures, there are fewer similar studies in Chinese society (Cheung and Wu, 2012). Our study complements earlier research by describing factors that promote successful aging in the Chinese context. In Oriental culture, the family field and the work field are highly coincident and will have an impact on older employees (Cho and Chen, 2018). Therefore, it is very important to consider the role of the family field in the Chinese context. Previous studies have focused on this and found that family-work conflict has a negative impact on successful aging at work (Cheung and Wu, 2012, 2013b), but did not pay attention to the impact of work-family enrichment on successful aging at work. Our study is an extension of previous studies. Our data show that both W-FE and F-WE have a positive effect on SAW. Secondly, the antecedent research of SAW is expanded from the perspective of work-family enrichment. As mentioned above, SAW is very important in practice, and scholars have explored its causes extensively. However, most of the existing studies attribute the causes of SAW to the work field, ignoring the influence of family factors, which is a major shortcoming in the Chinese context where work and family frequently infiltrate each other (Zacher et al., 2018). The mutual penetration of work and family includes two aspects of work-family conflict and work-family enrichment, which can affect employees’ behavior (Zhang et al., 2018; Mishra et al., 2019). Therefore, when analyzing the causes of SAW of older employees, it is not sufficient to consider only the work factor without considering the mutual penetration of work and family. Based on the above analysis, the first theoretical implication of this study is that based on the general cognition that employees’ positive behaviors are mostly derived from positive influencing factors (Alnaimi and Rjoub, 2021), according to the COR, this study analyzes the antecedents of SAW from the positive aspects of work-family penetration, that is, from the perspective of work-family enrichment. The research conclusions not only make up for the shortcomings of previous studies that only consider the antecedents of SAW from the work field but also respond to the suggestion that scholars should consider how to stimulate the positive behavior of older employees from the perspective of the family field (Cheung and Wu, 2013a). Thirdly, scholars believe that work-family enrichment should include the connotation of size, direction, and symmetry (Allen et al., 2014). However, most scholars still analyze its impact from a single perspective. This study fully considers the direction of work-family and analyzes the two-way influence of W-FE and F-WE. The research conclusions enrich the cognition of the directional characteristics of work-family enrichment in empirical research and provide some reference for future research. Fourthly, the moderate role of AIHRP between W-FE, F-WE, and OFTP was explored. As far as we know, the role of AIHRP as a moderating variable in the relationship between OFTP and its antecedents has received little attention. By demonstrating the promoting effect of AIHRP, we can gain an in-depth understanding of the boundary conditions for AIHRP to create a favorable working environment for older employees. Our analysis found that high levels of AIHRP help to enhance the effect of F-WE/W-FE on OFTP. One possible explanation is that age bias and age discrimination in the organization affect the use of resources generated by work-family enrichment by older employees (Oliveira, 2021), and the intervention of AIHRP helps older employees to better apply family resources to work. Older employees perceive inclusion and respect, the organization provides guarantees for the impact of work-family enrichment on the work field, and lays the foundation for older employees’ broader OFTP (Oliveira, 2021; Rudolph and Zacher, 2021), our research extends the study of age-inclusive HR practices in the work-family domain. Finally, in the Chinese context, the mediating role of OFTP between work-family enrichment and SAW is demonstrated. There are few studies on SAW in the Chinese context, and family has a unique significance for Chinese people. This study explores the two-way influence of W-FE and F-WE in the Chinese context, enriches the relevant research of OFTP, and makes up for the mechanism of work-family enrichment on SAW. Consistent with previous studies, it was found that OFTP was positively correlated with SAW (Cheung et al., 2019). The results of the mediation model show that F-WE predicts SAW by increasing OFTP, and W-FE predicts SAW by increasing OFPT. In other words, the gain resources of F-WE/W-FE help to improve the OFTP of older employees. Older employees who experience F-WE/W-FE break the shackles of resources and have a broader view of their future development. Previous studies have found that older employees have a stronger positive relationship between W-FE and OFTP (Henry et al., 2018), and our findings support this finding (Jung Choi and Tae Kim, 2012; Zhang et al., 2018). We find that F-WE is positively correlated with work-related variables, which can also be explained by cross-domain effects (Zhang et al., 2018). The mediating role of OFTP in family relationship and work-result relationship further enriches the relationship between F-WE and work-related variables (Alcover et al., 2022). In addition, OFTP (perceived remaining time, focus on opportunities, focus on limitations) with three dimensions is becoming more and more effective and common (Zacher, 2013; Topa and Zacher, 2018; Alcover et al., 2022). Future research can use three-dimensional measurement to study OFTP. It provides a reference for explaining the mechanism of work-family enrichment on the behavior of older employees in the Chinese context.

### 6.2. Practical implications

We get some practical implications from this study. First, according to the conclusion that the higher the work-family enrichment, the higher the OFTP of older employees, this suggests that managers should promote W-FE and F-WE of older employees. On the one hand, organizations and older employees should form a concept of mutual support. At the organizational level, it can create an atmosphere where older employees can get fair and equal returns and implement AIHRP. At the employee level, organizations can advocate to employees their commitment to meeting the needs of older employees and their families, creating awareness among older employees that they should be reciprocal with the organization. On the other hand, organizations and employees should be able to support each other. For example, organizations need to provide family support for employees, such as child care and a flexible work system, so that older employees and their families can get corresponding resource support when needed. At the employee level, it is also necessary for older employees to promise to provide family resources to support the organization, such as requiring the family to allow older employees to work overtime due to emergencies. Second, because OFTP can promote the conclusion of SAW, it suggests that managers should try to improve the OFTP of older employees. In specific practice, on the one hand, organizations can provide resources to help older employees deal with family affairs, encourage their families to support employees’ work needs, and help older employees have open future career practices and opportunities. On the other hand, organizations can improve the ability of older employees to deal with work and family affairs efficiently through training. For example, by reasonably arranging their task lists and focusing on investment, employees can be prompted to deal with work and family affairs efficiently, thus better balancing the needs of family and work. In addition, we believe that our research results can be further promoted in Greater China, and may even be promoted in other Asian countries such as South Korea or Japan, because they all have “family culture” and high collectivism (Oyserman et al., 2002).

### 6.3. Limitations and future research

First, in terms of research design, although this study adopts the method of collecting data at multiple time points in the way of data collection because the number is derived from the self-report of employees, the common method deviation problem is not serious, but it is still unavoidable. Therefore, future research should try to increase the multi-source of data sources. In addition, the locations and enterprises of data collection are also limited. Future research can expand the sampling scope and consider the randomness of the target locations and enterprises to increase the universality of the conclusions. There is not a longitudinal approach over time (aging) to analyze the process of success. To better understand the dynamic processes and outcomes of successful ageing at work (Zacher et al., 2018), future research should use longitudinal design to examine the relationship between the work-family interface and successful ageing in the workplace. It will be interesting to consider a longer time frame in future studies, as these effects may take more than 18 months to show a different trajectory of change. Finally, although this study considers the direction of work-family enrichment, it does not consider the impact of its symmetry (Allen et al., 2014). Future research can be based on the symmetry of work-family enrichment.

## Data availability statement

The original contributions presented in this study are included in the article/supplementary material, further inquiries can be directed to the corresponding author.

## Ethics statement

Ethical review and approval was not required for the study on human participants in accordance with the local legislation and institutional requirements. Written informed consent for participation was not required for this study in accordance with the national legislation and the institutional requirements.

## Author contributions

CZ organized the database and wrote some of the manuscript. HM statistically analyzed the data and wrote the first draft of the manuscript. ZC collected the data, drafted and revised the manuscript. XL analyzed the data, drafted and revised the manuscript. All authors contributed to the conception and design of this study and were involved in revising and reading the manuscript.
